# Implications of initial physiological conditions for bacterial adaptation to changing environments

**DOI:** 10.15252/msb.20209965

**Published:** 2020-09-23

**Authors:** Matthias Heinemann, Markus Basan, Uwe Sauer

**Affiliations:** ^1^ Molecular Systems Biology Groningen Biomolecular Sciences and Biotechnology Institute University of Groningen Groningen The Netherlands; ^2^ Institute of Molecular Systems Biology ETH Zurich Zurich Switzerland; ^3^ Department of Systems Biology Harvard Medical School Boston MA USA

**Keywords:** Metabolism, Microbiology, Virology & Host Pathogen Interaction

## Abstract

This piece discusses how the different observations of two independent studies (Kotte *et al*, 2014; Basan *et al*, 2020), regarding population‐level heterogeneity and lag times during diauxic shift, can be largely explained by different experimental protocols.

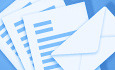

Overcoming metabolic transitions is a key determinant of microbial fitness. Some of the most challenging transitions are encountered when microbes are shifted from rapid, often partly fermentative glycolytic to slower, fully respiratory gluconeogenic growth with lag phases that can last many hours without detectable biomass production. Our groups have recently worked extensively to understand the mystery how such long lag phases emerge and why they cannot be easily overcome by bacteria.

Our independent studies Kotte *et al* (2014) and Basan *et al* (2020) appear at first glance somewhat contradictory, because we observed very different phenomenologies, regarding population‐level heterogeneity and also the extent of observed lag times. While Kotte *et al* (2014) found distinct subpopulations of cells, with some cells that grew immediately and others that never resumed growth, Basan *et al* (2020) found a unimodal distribution of single cell lag times and the vast majority of cells eventually resumed growth. The resulting lag times from Kotte *et al* (2014) were also much longer than those observed in Basan *et al* (2020).

These differences are largely the result of different experimental protocols for performing the metabolic shifts. In Basan *et al* (2020), bacteria were washed twice on a filter with warmed post‐shift medium and then gently resuspended with a pipette in warmed medium, a procedures accomplished in 2–3 min. Thus, cells were instantaneously exposed to the new substrate and lag phases were only a few hours, closely resembling the apparent phases of no growth during the diauxic shifts in batch experiments (Erickson *et al*, 2017; Basan *et al*, 2020). In contrast, the protocol of Kotte *et al* (2014) included several rounds of centrifugation and washing with ice‐cold, substrate‐free medium. The entire procedure took about 20 min and likely exposed the bacteria to starvation and cold stress. Perhaps not surprisingly, the protocol by Kotte *et al* (2014) resulted in much longer lag times and a different phenomenology. Hence, as illustrated in Fig 1, cells in the two experiments started their adaptation from very different initial physiological conditions. While the “Basan” cells presumably had to mainly reverse their glycolytic flux, the “Kotte” cells likely faced additional challenges that might include a more severe drop in energy supply and additional adaptations of their proteome during the washing phase.

**Figure 1:**
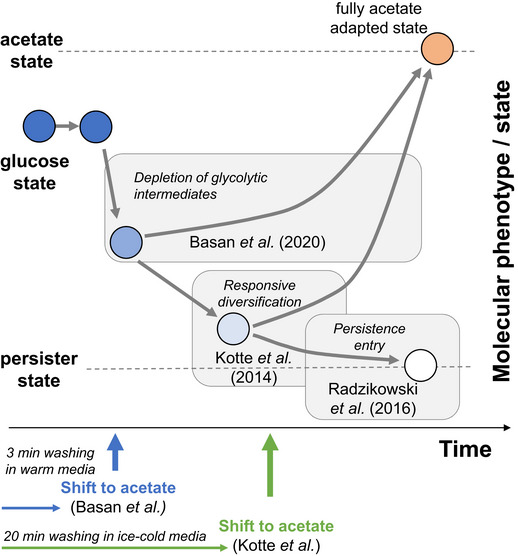
**Schematic of phenotypic states and relevant molecular challenges for the diauxic shift from growth on glucose to acetate of** ***Escherichia coli***.

Independent of the physiological starting point, cells in both experiments had to overcome the problem of depletion of key glycolytic metabolites that result from the sudden flux reversal when switching from glycolytic to gluconeogenic growth. This imposes a trade‐off between rapid growth and adaptability that was also observed in Kotte *et al* (2014), caused by sequential flux limitation in gluconeogenesis (Basan *et al*, 2020). Imposing additional stress during the transition phase can drive the population into subpopulation heterogeneity, which can be observed as an increased length of the lag phase (Kotte *et al*, 2014). Thus, rather than contradicting each other, our studies offer a complementary picture of the physiological adaptation from glycolytic to gluconeogenic growth.

Beyond the specific case of understanding the challenges of the diauxic shift, our results highlight the critical importance of the precise environmental conditions prior to introducing any physiological change or starting a measurement. Seemingly small differences such as placing cells on ice, washing in nutrient‐free buffer, or length of the procedure can rapidly alter the physiological state of the culture and/or its population structure. Precise reporting of these seemingly trivial procedures is therefore mandatory and must be taken into account when comparing data from different sources.
